# Does Industrial Agglomeration or Foreign Direct Investment Matter for Environment Pollution of Public Health? Evidence From China

**DOI:** 10.3389/fpubh.2021.711033

**Published:** 2021-08-19

**Authors:** Shi-Jie Li, Bin Sun, Ding-Xia Hou, Wei-Jian Jin, Yun Ji

**Affiliations:** ^1^School of Economics, Hainan University, Hainan, China; ^2^Business School, Foshan University, Foshan, China; ^3^Academy of Financial Research, Business School, Wenzhou University, Wenzhou, China

**Keywords:** industrial agglomeration, foreign direct investment, environmental pollution, public health, threshold

## Abstract

This article focuses on the interaction between China's industrial agglomeration, foreign direct investment (FDI) and environmental pollution of public health in the past 15 years. By conducting theoretical and empirical research, we try to reveal the relationship and mechanism between the economic growth and public health from the perspective of environmental pollution. By constructing an embedded theoretical model of industrial agglomeration and FDI, this article combines other environmental pollution influencing factors, expounds the impact mechanism of industrial agglomeration on environmental pollution. Based on the provincial-level panel data of China on environmental pollution and industrial agglomeration, the empirical test is carried out through the threshold panel regression model. According to the results, industrial agglomeration can significantly rectify the regional environmental pollution, thereby benefiting public health. FDI has a phased impact on the relationship between industrial agglomeration and environmental pollution. Specifically, when the level of FDI is low, the positive improvement effect of industrial agglomeration on environmental pollution is relatively strong. This is mainly because industrial agglomeration can promote economic growth, technological progress, and enhance environmental awareness. When the level of FDI exceeds the first threshold and continues to rise, the positive improvement effect of industrial agglomeration is maximized. Before the level of FDI exceeds the second threshold, this effect gradually weakens. The population concentration and excessive expansion of city scale brought about by industrial agglomeration will lead to the increase of regional resource and energy consumption, thus aggravating environmental pollution. The policy implication is that while the government and enterprises are vigorously increasing the level of foreign investment, they must pay equal attention to economic growth and public health, and the level of industrial agglomeration should match the level of foreign investment so as to give full play to the positive improvement effect of industrial agglomeration on environmental pollution, and realize the coordinated development of the regional economy, environment and population health.

## Introduction

The ultimate goal of economic development is to improve the public welfare. However, as the history of human development has proved, the process of economic growth cannot fully achieve this goal. The environmental Kuznets Curve indicates a trade-off relationship between income increased and ecological environment (1). Economic growth may lead to environmental degradation, bring harm to public health, and therefore undermine the public welfare (2). Take China as an example. In the past four decades, China has indeed achieved rapid economic growth, but this has been accompanied by serious environmental pollution, which has severely damaged public health (3, 4). For example, in 2017, the substandard rate of 338 air quality monitored cities in China greatly exceeds 70%. Therefore, from the perspective of environmental pollution, an important transmission mechanism between economic growth and public health can be revealed.

Based on a typical fact that industrial agglomeration and FDI plays an important role in the process of Chinese economic development, combined with the trend of ecological environment change, this article attempts to simultaneously examine the complex interaction of industrial agglomeration and foreign direct investment (FDI) on environmental pollution empirically. Through this special mechanism, we can reveal the possible negative effects of economic growth on public health, and expounds the methods to reduce environmental pollution in an effort to bring broader benefits to public health. Consequently, this article joins the discussion about the relation among economic development, environment quality, and public health in the recent literatures. Using Dual-track approach to measure citizen mobility in Italy, De Maria et al. (5) found that during the lockdown of the new coronavirus in Italy, while the Italian government restricted population movement and economic growth, the urban pollution was greatly reduced. Research results of Sun et al. (6) shows that economic development can increase public expenditures for the prevention and treatment of foreign infectious diseases, which is conducive to protecting public health. Pu et al. (7) used the panel data of 31 regions in China from 2002 to 2018 and concluded that economic development has reduced medical expenditures, and unfair income distribution will exacerbate such effects (7). Su et al. (8) applied panel threshold regression model in BRICS and ASEAN countries to conclude that overheated economic growth will have a negative impact on the medical and health system and is detrimental to public health (8). These studies obtained different and even mixed results. Our study can contribute this field by revealing the channel connecting industrial agglomeration, FDI, and environmental pollution.

Since the reform and opening up of China, FDI has continued to grow, and industrial agglomeration has driven rapid economic development. However, environmental pollution has gradually aggravated to be a simultaneous side effect. Some of the studies carry out empirical studies, and draw conclusions that industrial agglomeration and environmental pollution are, respectively, positively correlated, negatively correlated, and non-linearly correlated (9–11). With respect to the impact of FDI on environmental pollution, there are two contradictory arguments in the existing literatures, namely, hypothesis of “pollution paradise” and hypothesis of “pollution halo” (12). When industries are transferred through FDI, it is usually accompanied by the introduction of knowledge, technologies, and management methods. The transfer of industrial production and the diffusion of knowledge and technology not only fuel the development of industrial agglomeration, but also exert some negative impacts on the local environment of the host country. In addition, the promoting effect of FDI on industrial agglomeration worsens the environmental pollution to some extent. This means that there are some connections between these aspects to a certain extent. However, the nature and direction of these relations are still unclear. The FDI can directly or indirectly affect the environmental effects of industrial agglomeration. Since environmental quality is directly related to public health (13), there is an urgent need for an in-depth study on the complex interaction and transmission mechanism between them.

Based on the above reasons, this paper constructs theoretical models of FDI, industrial agglomeration, and environmental pollution using a scalable random environmental impact assessment model. According to the model, FDI will have a positive or negative influence on the relation between industrial agglomeration and environment. In the context of FDI, there is a non-linear correlation between industrial agglomeration and environmental pollution. Therefore, it is hereby proposed to adopt Panel Threshold Measurement Model for empirical testing. Based on 14-year panel data from 30 provinces and cities (includes autonomous regions) in China, we examined the environmental impact of industrial agglomeration at different levels of foreign investment, and objectively evaluates the environmental effect of FDI and agglomeration of industries. On the one hand, this article reveals an important transmission mechanism between economic growth and public health and makes theoretical contributions to related fields. On the other hand, it also puts forward the policy recommendations to promote the coordinated development of the economy, environment, and public health of China.

## Literature Review

In order to clearly unfold a big picture of relevant literatures in this field, this section reviewed the research about the relationship among economic growth, environmental pollution, and public health, as well as the relationship among industrial agglomeration, FDI, and environmental pollution. Comments will be provided in some places, if necessary.

### Economic Growth, Environmental Pollution, and Public Health

The relationship between economic growth and environmental pollution is usually expressed as an Environmental Kuznets Curve (14). As shown by this curve, at a certain stage of development, income growth driven by economic growth cannot be combined with the protection of the ecological environment. It is necessary to carefully weigh the pros and cons in order to make the final choice. For example, places with severe environmental pollution are more likely to attract capital, labor, and other resources due to the relatively relaxed environmental regulation threshold, which will enable them to vigorously develop their economy and increase the income of the residents. However, the deterioration of the ecological environment, caused by economic development, seriously jeopardizes public health. Studies have shown that most Chinese families cannot afford medical care without major health insurance (15). As a result, when residents feel intolerant to the local ecological environment, they often choose to relocate to other places, thereby forcing local governments to make concessions at the expense of economic growth to some extent and improve the ecological environment of their jurisdictions (16). Chen and Xiao (17) added multiple factors (such as environmental pollution) to Grossman's Production Function of Health, and adopted panel data of 30 Chinese provinces and municipalities (autonomous regions) from 1997 to 2010 to conduct an empirical analysis (17). They found that there is a long-term and balanced cointegration relationship among economic growth, environmental pollution, medical and health services, and public health of residents, while the relationship between economic growth and public health is characterized by an inverted U-shape from the perspective of the entire country and the eastern and central regions. Therefore, policymakers must weigh the pros and cons among environmental quality, public health, and economic development (18).

Many empirical facts and academic research have proved the threat of environmental pollution to public health. In the last century, the world has been hit by many serious hazardous incidents to health, which are caused by environmental pollution, such as London Smog Incident and Los Angeles Photochemical Pollution Incident. These incidents posed huge threat to public health. A qualitative study by (19) proves that carbon monoxide has a significant catalytic effect on the morbidity rate of asthma in children aged 1–18 (19). Air pollution has led to a decline in the human lung function, worsening of the morbidity and mortality of bronchitis, pulmonary heart disease, and respiratory diseases, and an increase in the number of inpatients and outpatients even aggravates the premature deaths of residents (4). Through quantitative researches, Dales et al. (20, 21) believed that air pollution, water pollution, or other pollution types of environmental pollution will harm the health conditions of residents (20, 21). The degree of harm caused by environmental pollution to public health also depends on the degree of pollution exposure. People who have been living in heavily polluted areas for a long time are more likely to get sick (22).

The process of the economic development of China has also been stained by frequent public health damages due to environmental pollution. According to statistics, among the 232 major environmental incidents that occurred during the “Eleventh Five-Year Plan” period, there were 56 public health damage incidents caused by environmental pollution (13). According to Zhao et al. (23, 24), the energy structure of China, which is dominated by the consumption of coal-fired energy and other fossil energy, is the primary cause of severe air pollution (23, 24). With reference to this point, a study uses the Greenhouse Gas and Air Pollution Interactions and Synergies (GAINS) model to predict that China could reduce carbon dioxide emissions by 32 percent by 2030, if it actively adopted renewable energy (25). Based on the Long-range Energy Alternatives Planning (LEAP) Model and Exposure Response Model, some researches further concluded that under strict energy conservation control, a decrease in the proportion of coal consumption reduces the corresponding mortality ratio (26, 27). According to the empirical research by (3), from the perspective of the whole country and the eastern, central, and western regions, the emission load of industrial fumes is positively correlated with population mortality rate, and emission load of industrial sulfur dioxide are significantly positively correlated with population mortality rate in the central region (3). In general, environmental pollution and ecological destruction have caused serious economic and social losses to China. The annual loss due to environmental pollution accounts for about 10% of the GDP of China. Every year, 213,000 patients suffer from pulmonary heart disease and 1.5 million suffer from chronic bronchitis due to air pollution (28). The disease burden of residents, caused by environmental pollution, accounts for ~21% of the total disease burden (3). Therefore, further revealing the ways and transmission mechanisms of economic growth affecting public health through environmental pollution has important theoretical value and practical significance. From a new perspective, this paper conducts an empirical study on the complex interaction among industrial agglomeration, FDI, and environmental pollution, and provides a useful reference for policymakers.

### Industrial Agglomeration, FDI, and Environmental Pollution

Regarding the relationship between FDI and industrial agglomeration, Amiti and Javorcik (29) believed that in case of improved freedom of trade in a country, upstream and downstream enterprises often go with the stream and swarm into this country (29). The FDI promotes the agglomeration of local industries by improving the technological innovation capacity of the host country and realizing technological spillover of such capacity among enterprises (30). Other academic literature also pointed out that FDI characterized by movement following certain regional policy advantages stimulates the increase of regional FDI, thereby providing impetus for regional industrial agglomeration (22, 31).

Regarding the impact of industrial agglomeration on environmental pollution, researchers in this field have different opinions. The research findings, which hold “aggravation of environmental pollution,” indicate that negative externalities of industrial agglomeration, that is, the concentrated discharge of pollutants will lead to the deterioration of the environment in the agglomeration areas (9). This negative effect is not only apparent in the developing countries, but also in the developed countries (32, 33). In addition, there may also be a two-way mechanism between industrial agglomeration and environmental pollution. In other words, the more serious the environmental pollution, the greater the driving force for industrial agglomeration (34). On the contrary, the research findings, which hold “suppression of environmental pollution,” indicate that industrial agglomeration may have an adverse impact on the environment in the short term, but in the long run, it will also bring many benefits to the agglomeration areas. Manufacturing industry agglomeration can alleviate the effect of “Pollution Paradise” (10). Shen et al. (35) believed that industrial agglomeration reduces the cost and risk of low-carbon innovation and increases carbon productivity by promoting the spillover and sharing of environmental protection and energy conservation knowledge among enterprises in industrial agglomeration areas (35). Li et al. (36) applied panel data vector autoregressive model to analyze the dynamic relationship between industrial agglomeration and environmental pollution and revealed that the degree of industrial agglomeration can significantly reduce industrial pollution emissions (36). There is no doubt that many research results also show the non-linear relationship between the two, which may be affected by other factors (11, 37).

Regarding the relationship between environmental pollution and foreign investment in a region, there have been two completely different hypotheses in academia for a long time, namely “pollution paradise” and “pollution halo.” Walter and Ugelow (38) initially put forward the hypothesis of “pollution paradise,” which pointed out that the transfer of pollution from the home country is the main reason for the foreign companies to enter the local market (38). The increase in polluting enterprises, triggered by FDI, will result in the ever-increasingly poorer environmental quality of the host country. Hypothesis of “pollution halo” takes the opposite view, and states that the entry of foreign capital into the local market can bring about environment-friendly and efficient technologies. Through technology spillover effects, the overall technological level of region-wide enterprises will rise, the production efficiency of local enterprises will be improved, the emissions of enterprises will be reduced, and the local environmental quality will turn for the better. For example, a study points out that multinational companies can promote the technological upgrading of local enterprises and the improvement of population quality through labor mobility and technology spillover, thereby affecting the local environmental quality (39).

In fact, hypothesis of “pollution halo” is more obvious in the Chinese context. Li et al. (11) conducted an empirical analysis and proved that the main reason for reducing air pollution in the manufacturing industry is FDI and other external factors, but the internal motivation for energy conservation and emission reduction is insufficient (12). Yang (40) adopted the panel data of 227 Chinese cities from 2004 to 2012 to empirically analyze the relationship between industrial agglomeration and environmental pollution (40). The analysis results show that when the level of FDI is low, industrial agglomeration exacerbates environmental pollution. When the level of FDI is high, industrial agglomeration alleviates environmental pollution. Su et al. (41) used the panel cointegration analysis method to verify the hypothesis of “pollution paradise” and the hypothesis of “pollution halo.” They found that if capital intensity continues to go up, pollution coefficient of FDI partly declines (41).

With reference to the above-mentioned related literatures, it is found that there is a clear correlation among industrial agglomeration, FDI, and environmental pollution, whereas environmental pollution poses a threat to public health. Furthermore, as a channel, FDI has a say in the relationship between industrial agglomeration and environmental pollution. Therefore, when studying the impact of industrial agglomeration on the environment, FDI, which is an important factor, cannot be ignored. However, the existing research findings only use the linear regression model as the main measurement model analysis strategy, which fails to portray the staged impact of industrial agglomeration on environmental pollution on a profound dimension. Therefore, this paper attempts to introduce the “Panel Threshold Regression Model” proposed by Hansen, taking into account the domestic panel data at the provincial level, and examines the impact mechanism and channels of industrial agglomeration on environmental pollution at different levels of FDI. This paper further reveals the inner connection between industrial agglomeration and environmental pollution, in order to provide a theoretical explanation for the contradictory between industrial agglomeration and environmental pollution, and gives useful references for policymakers to formulate policies in connection with public health.

## Methods

### Theoretical Analysis and Research Hypotheses

Based on the Copeland–Taylor General Equilibrium Theory Model, aiming at the technological spillover and technological innovation effects brought about by industrial agglomeration and foreign direct investment, this paper leverages the inherent technological factors in the classical production function, and integrates industry agglomeration, FDI, and environmental pollution, in an effort to construct a theoretical model on the impact of industrial agglomeration and FDI on environmental pollution, and probe into its relationship with public health on these grounds.

### Production Function

Suppose that in an economic system, only non-polluting products, *Z* and capital-intensive polluting products, *X* are manufactured. At the same time, industrial enterprises will emit pollutants, *Y* that endanger environmental quality during the production process of product *X*. Degradation of environmental quality, caused by the discharge of pollutant, will have a negative external effect on the society as a whole. Therefore, emission of pollutant *Y* incurs social cost. According to Coase Theorem, if property rights are specific and trading cost is enough low or zero, such externality can be resolved through market mechanism, manifested as pollution rights trading or pollution charges. This also means that companies will use certain production factors for environmental governance as the cost of pollutant discharge. If the ratio of the production factors applied by the enterprise for pollution control to the total production factors is γ, in this case, 0 ≤ γ ≤ 1. In other words, if γ = 0, the enterprise does not control pollution, and in this case, the actual output is the potential output, *F*. If γ = 1, the enterprise will use all production factors to control pollution, and the actual output is zero in this case. If 0 < γ <1, production factor, γ*F* is used to control pollution, and in this case, the actual output is (1-γ) *F*, and pollutant *Y* will appear simultaneously. According to the above expression, the equations are specified as follows:

(1) $$\begin{array}{l} X=\left(1-\gamma \right)F \end{array}$$

(2) $$\begin{array}{l} Y=\psi \left(\gamma \right)F \end{array}$$

(3) $$\begin{array}{l} \text{Ψ}\left(\gamma \right)=\frac{1}{A}{\left(1-\gamma \right)}^{\frac{1}{\alpha }} \end{array}$$

Where, ψ(γ) is a decreasing function of γ, that is, ψ′(γ) < 0 and ψ″(γ) > 0. *A* indicates production technology, α is a parameter, and α ∈ (0, 1). Function *F* is a homogeneous production function and complies with the property of constant returns to scale of the production function.

If the production factors only include capital *K* and labor *L*, the above production function can be expressed as follows:

(4) $$\begin{array}{l} X=\left(1-\gamma \right)F\left(K_{X},L_{X}\right) \end{array}$$

(5) $$\begin{array}{l} Y=\text{Ψ}\left(\gamma \right)F\left(K_{X},L_{X}\right) \end{array}$$

(6) $$\begin{array}{l} Z=G\left(K_{Z},L_{Z}\right) \end{array}$$

After Equation (3) is introduced into Equation (5):

(7) $$\begin{array}{l} Y=\frac{1}{A}{\left(1-\gamma \right)}^{\frac{1}{\alpha }}F\left(K_{X},L_{X}\right) \end{array}$$

(8) $$\begin{array}{l} X={\left(AY\right)}^{\alpha }F{\left(K_{X},L_{X}\right)}^{1-\alpha } \end{array}$$

Equation (8) implies that output of product *X* depends on the input of pollutant *Y* and potential output *F*, and the production function of X satisfies the characteristic of constant returns to scale. The value of α indicates the ratio of effective pollution to total production cost; and 1-α indicates the percentage of potential output in the total production cost.

Driven by the instinct driven by the pursuit of profit, market enterprises will inevitably measure the production benefits and pollutant emission costs of products in the production decision-making process, and determine the appropriate output and pollution emissions. An analysis of the choice is made by a profit-oriented enterprise in the production decision-making and pollutant discharge decision-making.

### Production Decision-Making

Under the principle of profit maximization, every enterprise should work hard to “minimizing the cost while maintaining the same output” or “maximizing the output while maintaining the same cost.” This paper herein takes the former as an example to decide on the production of product *X*. From Equation (8), it can be seen that the production of capital-intensive product *X* of industrial enterprises is related to pollutant emission *Y* and potential output *F*. Therefore, when deciding on the production of product *X*, it is advised to follow the principle of “minimizing the cost while the output remains unchanged,” and clearly specify the minimum production cost *C*_*F*_ of unit potential output *F* under a given capital cost *r* and labor cost *w*. It is further found that where pollution control cost is λ, the minimum production cost*C*_*F*_of the unit product *X* is made clear, as shown in the following equations:

(9) $$\begin{array}{l} C_{F}\left(r,w\right)=\min\left\{rk+wl,F\left(K_{X},L_{X}\right)=1\right\} \end{array}$$

(10) $$\begin{array}{l} C_{F}\left(r,w\right)=\min\left\{\lambda AY+C_{F}F,\;{\left(AY\right)}^{\alpha }F^{1-\alpha }=1\right\} \end{array}$$

Under optimization solution, it can be seen from Equation (9) that when *C*_*F*_is the minimum, the marginal rate of substitution of capital and labor is equal to the ratio of labor cost to capital cost, as shown in the following equation:

(11) $$\begin{array}{l} TRS_{LK}=\frac{F_{L}^{\prime }}{F_{K}^{\prime }}=\frac{w}{r} \end{array}$$

Under optimization solution, it can be seen from Equation (10) that when *C*_*X*_ is the minimum, the marginal rate of the substitution of the potential output and effective pollutant discharge is equal to the ratio of the cost of pollutant discharge to the cost of potential output, as simplified in the following equation:

(12) $$\begin{array}{l} \frac{\alpha }{1-\alpha }\cdot \frac{F}{AY}=\frac{\lambda }{C_{F}} \end{array}$$

### Pollutant Discharge Decision-Making

It is assumed that the market in which an enterprise conducts business, is a perfectly competitive market, and *P*_*X*_indicates the price of product *X* subject to supply and demand in the market. The profit enterprise is zero, as shown in the following equation:

(13) $$\begin{array}{l} P_{X}=C_{F}F+\lambda \left(AY\right) \end{array}$$

According to Equation (12), *C*_*F*_*F* is expressed as λ(AY), and substituted into Equation (13), and pollutant emissions from the production of capital-intensive polluting products can be calculated as follows:

(14) $$\begin{array}{l} Y=\frac{\alpha P_{X}X}{\lambda A} \end{array}$$

When a non-polluting product *Z* is added to the economic system, then equation (14) can be changed into:

(15) $$\begin{array}{l} Y=\left(P_{X}X+P_{Z}Z\right)\frac{\alpha }{\lambda A}\frac{P_{X}X}{P_{X}X+P_{Z}Z} \end{array}$$

Where, *P*_*X*_*X*+*P*_*Z*_*Z* indicates industrial scale, which is substituted by *Sca*; and $\frac{P_{X}X}{P_{X}X+P_{Z}Z}$ refers to industrial structure, which is substituted by *Str*. Equation (15) can be changed into:

(16) $$\begin{array}{l} Y=Sca\cdot \frac{\alpha }{\lambda A}\cdot Str \end{array}$$

Equation (16) indicates that discharge of pollutants is negatively correlated with pollution emission cost, λ and technological level *A*, and positively correlated with the industrial scale, *Sca* and industrial structure, *Str* in a region. After logarithm is taken from the left and right sides of Equation (16), the following equation is obtained:

(17) $$\begin{array}{l} \ln\;Y=\ln\;\alpha +\ln\;Sca+\ln\;Str-\ln\;\lambda -\ln\;A \end{array}$$

The industrial scale and the industrial structure of agglomeration areas are subjected to the degree of industrial agglomeration (*agg*) and foreign direct investment (*fdi*), which accordingly have an impact on the emission of pollutants in the agglomeration areas. Industrial agglomeration is spatially manifested as the clustering of enterprises in a certain region, and industrial scale is expanded as a result. The entry of foreign enterprises through investment in host country enterprises or direct investment in local factories can bring in capital and capital inflows and help expand the industrial scale of agglomeration areas. Therefore, in this paper, the functional form of the industrial scale is set as follows:

(18) $$\begin{array}{l} Sca=\text{exp}\left(\beta_{0}+\beta_{1}\ln\;agg+\beta_{2}\ln\;fdi+\mu \right) \end{array}$$

The function of the industrial structure is set as follows:

(19) $$\begin{array}{l} Str=\text{exp}\left(\tau_{0}+\tau_{1}\ln\;agg+\tau_{2}\ln\;fdi+\delta \right) \end{array}$$

In industrial clusters, the spatial concentration of enterprises accelerates the flow of knowledge and professionals. Agglomeration drives technological innovation through technology spillover effects and competitive effects, and improves the overall technological level of the agglomeration areas. The FDI has introduced advanced technology and management methods. Due to the rapid flow of knowledge and professionals in agglomeration areas, technology spillover also plays a role and spreads in the agglomeration areas within a certain period. In addition, FDI has promoted technological innovation in the agglomeration areas through demonstration effects and competitive effects, thereby obtaining the improvement of production technology and environmental governance in the agglomeration areas. Therefore, in this paper, the function of technology is set as follows:

(20) $$\begin{array}{l} A=\text{exp}\left(x_{0}+x_{1}\ln\;agg+x_{2}\ln\;fdi+\upsilon \right) \end{array}$$

In the above equations (18), (19), and (20), *agg* indicates the level of agglomeration; *fdi* indicates the level of foreign direct investment.

Due to the parameter α, blowdown cost λ is exogenously given; they are not analyzed herein and collectively expressed as ξ_0_ together with the constant term. According to Equations (18), (19), and (20), the pollution emission intensity can be expressed as follows:

(21) $$\begin{array}{l} \ln\;Y=\xi_{0}+\xi_{1}\ln\;agg+\xi_{2}\ln\;fdi+\theta \; \end{array}$$

Where, ξ_0_ = ln α − ln λ + β_0_ + τ_0_ + *x*_0_, ξ_1_ = β_1_ + τ_1_ − *x*_1_, and ξ_2_ = β_2_ + τ_2_ − *x*_2_.

As can be seen from the above equation, the positive and negative properties of ξ_1_ and ξ_2_ are still uncertain. The impact of industrial agglomeration and FDI on the environment depends on the combined effect of industrial agglomeration and FDI through the industrial scale, industrial structure, and technological level of the positive and negative environmental externalities. There are complex correlations between industrial agglomeration, FDI, and environmental pollution. Therefore, our hypotheses are formally proposed as follows:

H1: Industrial agglomeration reduces environmental pollution, the higher the degree of industrial agglomeration is, the lower is the environmental pollution.
H2: FDi can change the relationship between industrial agglomeration and environmental pollution in terms of different threshold levels:
H2a: When FDI is either enough low, the negative effect of industrial agglomeration on the environmental pollution is weak.
H2b: When FDI is medium, the negative effect of industrial agglomeration on environmental pollution is strong.
H2c: When FDI is enough high, the negative effect of industrial agglomeration on environmental pollution is weak again.

## Measurement Model and Explanation for Variables

### Construction of Threshold Model

In this article, We refer to the panel threshold regression model proposed by Hansen (42), which is used herein. Based on the previous theoretical analysis, the level of foreign direct investment (fdi) is used as the threshold variable for the empirical analysis. The model settings are given as follows:

(22) $$\begin{array}{l} \ln\;con_{it}=\alpha_{i}+\beta_{1}\ln\;agg\cdot I\left(q\leq \lambda \right)+\beta_{2}\ln\;agg\cdot I\left(q>\lambda \right)\\ \;+\beta_{n}X_{it}+\varepsilon_{it} \end{array}$$

Where, *I*(·) indicates the indicative function, λ indicates the threshold value to be estimated, and the other variables are the same as described above.

In the single-threshold model, to verify whether there is a threshold effect and to further examine the significance of the threshold effect and the authenticity of the threshold estimate, firstly, null hypothesis *H*_0_ : β_1_ = β_2_ is made to conduct an empirical test of the threshold effect, in which the Bootstrap Method is applied to simulate the likelihood ratio test for asymptotic distribution of *F*-statistic and its critical value. If the *p-*value corresponding to the *F*-statistic is small (usually lower than 0.10), the null hypothesis is rejected, and the threshold effect is considered to exist. Otherwise, the null hypothesis will be accepted, and the threshold effect can be considered absent. Moreover, the likelihood ratio test for *LR*(λ) is used to calculate the confidence interval of λ and test the authenticity of the threshold value. Similar to the testing method under the single-threshold model, due to space limitations, this article will not repeat the testing of the dual-threshold model and the multi-threshold model.

### Variable Selection and Data Processing

**(1) Explained Variable:** The level of environmental pollution. The main influencing factors of environmental pollution include exhaust gas, wastewater, and solid waste. Taking into account the availability of data and the true reflection of environmental pollution, this article uses industrial sulfur dioxide, industrial waste water, and industrial oil fume emissions as the measurement indicators of the environmental pollution in various regions, and comprehensively reveals the environmental pollution in various regions. Among them, analytic hierarchy process (AHP) is applied to calculate the proportion of various pollutants in the overall environmental pollution through calculation and verification and refer to the method from (43) to determine the weights of various pollutants (43). The weights of industrial sulfur dioxide, industrial wastewater, and industrial fumes are found to be 0.36, 0.40, and 0.24, respectively. In this paper, comprehensive environmental pollution load is set as follows:

(23) $$\begin{array}{l} pol_{i}=\sum_{k=1}^{3}v_{k}{\overset{-}{x}}_{ik} \end{array}$$

Where *pol*_*i*_ represents the comprehensive environmental pollution load of the *i*-th city; ${\overset{-}{x}}_{ik}$ represents the discharge quantity of the *k*-th type of pollutant in the *i*-th city which has undergone dimensionless processing, and *v*_*k*_ indicates the weight of the *k*-th type of environmental pollutant. Another indicator is such a comprehensive indicator of environmental pollution, which indicates the degree of environmental pollution, expressed as the pollution load per unit output value:

(24) $$\begin{array}{l} con_{i}=\frac{pol_{i}}{{ȳ}_{i}} \end{array}$$

Where ȳ_*i*_ indicates the gross industrial output value of the *i*-th area that has undergone dimensionless processing.

Since *China Environmental Statistics* only disclosed the data on industrial fume pollutants from 2011 to 2016, the data of industrial fume emissions before 2011 can be obtained from *China Industry Statistical Yearbook* so as to calculate the level of environmental pollution in the major cities of the Chinese industrial agglomeration areas from 2004 to 2017.

**(2) Public Health:** Public health is the field of sociology related to the health of the people. Due to the large fluctuations in the absolute value of the number of deaths each year, it is not comparable to the absolute value. It is more practical to measure the level of public health by the ratio of the total number of deaths from respiratory diseases and lung diseases to the total number of deaths.

(25) $$\begin{array}{l} puh=\left(bre+lun\right)/mor \end{array}$$

Among them, *puh* is the level of public health, *bre* and *lun* are the death tolls of respiratory diseases and lung diseases, respectively, and *mor* is the total death toll of population. The death tolls of respiratory diseases and lung diseases are basically obtained from the “China Population Yearbook” and “Regional Population Yearbooks,” respectively. The data are true and reliable.

**(3) Core Explanatory Variables:** a. Industrial Agglomeration (*agg*): In the research design of this article, location entropy (*LQ*) is selected to measure the relative degree of spatial agglomeration in the industry. Due to the differences in labor productivity between or within industries, the statistical errors of employees in the informal economy and other factors are beyond human control; if the number of employees is used as a basic variable, the results will have less reference value. Therefore, in this paper, the actual gross industrial output value, which is converted with year 2004 as the base year and undergoes dimensionless processing, is used as the basic variable to calculate the location entropy, thereby avoiding incomparability due to the price level and the dimension difference between the variables in respective years. b. Foreign Direct Investment (*fdi*): In order to analyze how this intermediate variable affects the relationship between industrial agglomeration and environmental pollution, FDI is not only used as another core variable, but also as a threshold variable. In the industrial sector, the proportion of foreign investment in fixed assets can more effectively highlight the extent of FDI. In addition, to the availability of data, this article expresses the level of FDI as the ratio of foreign investment in fixed assets to the total FDI in the region.

(4) **Control Variables:** a. Technological Level (*tec*): It is an important indicator to measure the industrial production efficiency, environment friendliness, and environmental pollution control capabilities. It is expressed by labor productivity measured by the industry-wide gross product value. b. Industrial Structure (*str*): In this paper, this indicator is expressed as the ratio of the regional output value of the secondary industry to the regional GDP. c. Environmental Regulation Level (*reg*): This paper refers to the methods of previous studies (44, 45). This indicator is expressed as the ratio of the regional input in industrial pollution control to the national input in the industrial pollution control.

The calculation methods of each variable index are shown in Table 1. The basic data used in this paper are cited from China Industry Business Performance Data, China Statistical Yearbook, China City Statistical Yearbook, and other sources. Due to the lack of a large amount of Tibet Autonomous Region data, for the purpose of research work, it was finally decided to use panel data of 30 Chinese provinces and municipalities (autonomous regions), except Tibet. It should be noted that among the data of these 30 provinces and municipalities (autonomous regions) from 2004 to 2017, the data of individual regions or years are still missing. In this paper, interpolation method is applied to improve and supplement the missing data. Furthermore, this paper adopts the revised *Standard of National Industries Classification* (GB/T4754-2017) as the statistical criteria of the manufacturing industry.

**Table 1:** Specific economic meanings and calculation methods under panel regression model.

**Variable attribute**	**Variable name**	**Calculation formula**	**Explanation for variable meaning in the formula**
Level of Environmental Pollution	*con*	$con_{i}=\frac{pol_{i}}{{ȳ}_{i}}$	*pol*_*i*_: Comprehensive environmental pollution load. ȳ_*i*_: Standardized gross industrial output value.
Level of Industrial Agglomeration	*agg*	$agg_{i}=\sum_{j=1}^{n}\frac{x_{ij}/{\sum^{\text{​}}}_{j=1}^{n}x_{ij}}{x_{j}/{\sum^{\text{​}}}_{j=1}^{n}x_{j}}$	*x*_*ij*_: Gross industrial output value of the *i*-th industry in the *j*-th city. *x*_*j*_: Gross industrial output value of the *j*-th industry in the country. *n*: The number of industries in common.
Level of Industrial Technology	*tec*	$tec_{it}=\frac{y_{it}}{p_{it}}$	*y*_*it*_: Labor productivity calculated based on gross output value. *p*_*it*_: Consumer price index based on the price during base period of 1978.
Industrial Structure	*str*	$str_{it}=\frac{gdp_{2it}}{gdp_{it}}$	*gdp*_2*it*_: Gross output value of the secondary industry. *gdp*_*it*_: Gross output value of all industries.
Degree of Openness to the Outside World	*fdi*	$fdi_{it}=\frac{fgdp_{it}}{gdp_{it}}$	*fgdp*_*it*_: Total amount of foreign capital utilized in the society-wide fixed assets. *gdp*_*it*_: Amount of society-wide fixed assets investment in this region.
Intensity of Environmental Policy Regulation	*reg*	$reg_{it}=\frac{pg_{it}}{{\bar{pg}}_{it}}$	*pg*_*it*_: Total input in industrial pollution control. ${\bar{pg}}_{it}$: Mean value of the total input in industrial pollution control across all regions of China. (Total input in industrial pollution control includes the costs of exhaust gas, wastewater, and solid waste).

*In the above table, i, j, and t indicate a region, an industry, and a year, respectively*.

## Results

### Threshold Effect Test

Before estimating the threshold model, it is necessary to test whether there is a threshold effect and the significance of single threshold, double threshold, and triple threshold, and then verify the number of thresholds. After assuming single threshold, double threshold, and triple threshold in turn, the corresponding F value and *P*-value are obtained (Table 2). According to the results, the *P*-value corresponding to the value of the single-threshold F-statistic is 0.0000 and is significantly lower than 0.01, so the null hypothesis of the linear relationship between variables is rejected. Then the double threshold test is carried out. According to the results, the *P*-value corresponding to the value of the double-threshold F-statistic is still 0.0000, so the null hypothesis of a single threshold relationship between variables is also rejected. *P*-value corresponding to the value of the further triple-threshold F-statistic is higher than 0.10, and the null hypothesis of the double-threshold relationship between variables cannot be rejected. In short, the test results prove dual-threshold effect of the model. Under the influence of FDI, there is no single linear relationship between industrial agglomeration and environmental pollution.

**Table 2:** Threshold effect test.

**The number of thresholds**	**RSS**	**MSE**	**F value**	**P value**	**10% Critical value**	**5% Critical value**	**1% Critical value**
Single threshold	18.7556	0.0462	110.16	0.0000	35.8120	42.7288	55.8570
Double-threshold	16.2103	0.0399	63.75	0.0000	24.6645	29.6731	44.1526
Triple-threshold	19.6939	0.0485	11.61	0.1200	12.2799	24.7410	36.2274

The threshold values are further estimated. Under the confidence level of 95%, the estimated values of the double-threshold values are −3.4095 and −3.1978, respectively, and the confidence intervals are [−4.1291 to −3.4095] and [−7.0924 to −2.9015], respectively as shown in Table 3.

**Table 3:** The threshold estimation result.

**Threshold**	**threshold values**	**Left critical value**	**Right critical value**
“1–1	−5.3991	−7.0924	−2.9015
”2–1	−3.409469879	−4.1291	−3.4095
“2–2	−3.197808704	−7.0924	−2.9015

### Threshold Model Estimation

It can be seen from the threshold estimation result that there are two thresholds in this model. On this basis, we further analyzed the relationship between industrial agglomeration and environmental pollution within different FDI thresholds, and the impact of various control variables on environmental pollution. The estimated results of the panel threshold model are as shown in Table 4.

**Table 4:** Threshold and linear model estimation.

**Variable**	**Threshold Estimation**	**FE**	**SYS-GMM**
lnagg^*^I(q≦γ1)	−2.2145^***^		
	(−8.6486)		
lnagg^*^I(γ1 < q≦γ2)	−3.4455^***^		
	(−6.8035)		
lnagg^*^I(q>γ2)	−1.9292^***^		
	(−5.7364)		
lnagg		−0.5807779^**^	−1.136857^***^
		(−2.15)	(−5.48)
lnfdi		0.1697648^***^	0.0605472^***^
		(11.05)	(9.59)
lntec	−0.8821^***^	−0.4590924^***^	−0.2184677^***^
	(−12.7169)	(−6.39)	(−5.55)
lnstr	−1.5079^***^	−0.1471305	−1.182222^***^
	(−7.7661)	(-0.64)	(−13.76)
lnreg	0.0168	0.0385123^*^	0.0009014
	(0.6836)	(1.76)	(0.09)
cons	0.119965	0.3791804	−0.0006207
	(−0.2)	(0.90)	(−0.00)

The t or Z statistics are in parentheses in the table. “^***^”, “^**^”, “^*^” indicates significance levels of 1, 5, and 10% respectively. SYS-GMM is two-step.

The model has two thresholds, which are divided into three ranges according to the level of FDI. Within each scope, the degree of impact of industrial agglomeration on environmental pollution is different. Our hypothesis H2 is supported by the empirical evidence. In general, however, industrial agglomeration plays a role in inhibiting environmental pollution, and it is useful for improvement on public health. Our hypothesis H1 is supported by the empirical evidence. In addition, the technical level and industrial structure of industrial clusters are negatively correlated with environmental pollution, while environmental regulations are positively correlated with environmental pollution.

It can be seen from the results that in the three ranges of the level of FDI, the industrial agglomeration coefficient is strictly negative when the significance level is 1%. Specifically, when the level of FDI is lower than the first threshold value of 0.0331, the impact coefficient of industrial agglomeration on environmental pollution is −2.2145. In other words, agglomeration suppresses environmental pollution, and industrial agglomeration has a positive impact on the environment quality. Our hypothesis H2a is supported by the empirical evidence. When the level of FDI exceeds the first threshold but does not reach the second threshold of 0.0409, the impact coefficient of industrial agglomeration on environmental pollution is −3.4455. Our hypothesis H2b is supported. In other words, agglomeration suppresses environmental pollution, and industrial agglomeration has a positive impact on the environment. When the level of foreign investment continues to increase until it exceeds the critical value of 0.0409, the impact coefficient of industrial agglomeration on environmental pollution becomes −1.9292, which is still a negative value. Our hypothesis H2c is supported. Similarly, gatherings suppress environmental pollution, while industrial agglomerations have a positive impact on the environment.

From the analysis of the threshold regression results, it can be found that, in general, industrial agglomeration has an inhibitory effect on environmental pollution. After the positive externalities of industrial agglomeration offset the negative externalities brought about by agglomeration, there is still a “surplus,” which ultimately acts on the environment, restraining environmental pollution, and improving public health. However, at each stage of the FDI level, there is an interesting finding: the differences in the level of FDI can lead to different inhibition effects of industrial agglomeration on the environment. When the level of FDI is low, the environmental effect of industrial agglomeration is good. When the level of foreign investment rises, industrial agglomeration has the greatest restraint on environmental pollution, and industrial agglomeration has the greatest impact on the environment. When the level of foreign investment continues to rise and exceeds the limit, the inhibitory effect of industrial agglomeration on environmental pollution weakens, and the environmental effect of industrial agglomeration weakens. Therefore, only an appropriate level of FDI can effectively promote the environmental effects of industrial agglomeration.

When FDI is at a low level, foreign businesses enter and mainly transfer pollution-intensive industries into local economy, which adversely affects the local environment. With the increase in the level of FDI and the introduction of advanced production technology and management measures from abroad, due to the spillover effects of knowledge and technology, not only can the labor productivity of local enterprises be effectively increased, but also the utilization rate can be increased. Improving resource utilization and reducing pollutant emissions can also effectively improve local environmental governance capabilities. If FDI continues to grow beyond a certain limit, it will have a crowding effect. As foreign businesses seize the local enterprise market and plunge into a vicious competition, enterprises lay more emphasis on reducing costs and increasing output, and technological progress is also aimed to increase the output. High production causes high pollution. Moreover, “free-riding” happens in environmental governance, which will adversely affect the local environment and public health.

To ensure the authenticity and reliability of the empirical results, this paper estimates the linear model in equation (11). In consideration of the characteristics of the sample data in this paper and such a fact that Fixed Effect (FE) model can eliminate or control the unobservable and time-invariant individual heterogeneity and minimize the endogenous problem, the FE model is herein selected to regress the linear model. Compared with other methods, Generalized Method of Moments (GMM) does not require accurate distribution information of the random error terms, and can obtain more effective parameter estimates. At the same time, the system GMM is used for model estimation. For comparison, Table 4 summarizes the estimation results of FE and GMM. According to the estimation results, the coefficient signs of FE and GMM are the same, that is, the coefficients of industrial agglomeration are negative and significant at significance level of 5%. The coefficient of FDI is significantly positive. Technological level and industrial structure are significantly negatively correlated with environmental pollution at the significance level of 1%. However, the estimated results of the level of environmental supervision show that environmental pollution has increased. Through the analysis, it can be seen that the results of FE model test and GMM system are largely consistent with the results of the panel threshold regression. In this sense, the estimation results, obtained through panel threshold regression are reliable.

Looking back at the 14 years of historical experience of China in industrial agglomeration, pure industrial clusters will have a positive impact on the local environment if other factors remain constant. This is the result of the combined effect of many factors: Firstly, in the process of industrial agglomeration, knowledge and professionals converge. In the context of knowledge spillover, flow of professionals, and agglomeration market competition, technological innovation has become an inevitable choice. Technological progress contributes to the improved production efficiency of enterprises. Fixing the output as before, few raw materials need to be consumed and less pollutants emissions must be emitted. Secondly, in industrial agglomeration areas, input–output relation takes place in different types of enterprises. In other words, the by-products of one enterprise are the raw materials of another enterprise. This can avoid waste of resources, decrease resource consumption rate, and thus reduces pollution discharge rate. Thirdly, the expansion of industrial agglomeration scale will attract the attention of local government and public to the environment. A more well-established and strict environmental regulation system will be formed to strengthen the supervision of polluting enterprises and to levy penalties for violating enterprises. This will further boost the production and application of green technologies. If consumption of resources remains unchanged, more pollutants will be converted into clean resources for recycling and reuse of resources, thereby reducing the resource consumption and minimizing pollution emissions. Fourthly, although industrial agglomeration causes polluting enterprises in geographic space and centralized pollution emissions, the centralized pollution discharge will be resolved through centralized pollution control and other countermeasures. Centralized pollution control not only reduces the cost of pollution control, but also improves the efficiency of pollution control. These dividends generated by the agglomeration exceed the negative external influence of the agglomeration. Therefore, industrial agglomeration can effectively correct the status quo of environmental pollution, which is beneficial to public health, rather than exacerbating environmental pollution. This conclusion does not contradict with the earlier arguments that “industrial agglomeration aggravates environmental pollution” (33) and “industrial agglomeration inhibits and subsequently promotes environmental pollution” (46). The reason is explained herein: The industrial agglomeration of China has developed and formed under various economic, social, cultural, and other complex contexts. Therefore, scholars conduct analysis and research from different perspectives, and come to different conclusions.

As for other control variables, according to three regression results, technological level has a significant negative effect on environmental pollution at the significance level of 1%. In other words, since green production has become the mainstream of contemporary society, the high-tech field shows a low degree of environmental pollution. Moreover, the advancement of production technology cannot only greatly improve the economic benefits of the enterprises, but also improve the utilization rate of resources. If an enterprise prioritizes technological innovation, its economic efficiency and corporate image will be greatly embellished. As a result, the use of new technologies has attracted peer enterprises. The spillover effect of technology has prompted enterprises to successively introduce green and efficient technologies, thereby reducing regional environmental pollution. Technological advancement can also improve the regional environmental governance capabilities. Enterprises and local governments prefer centralized environmental governance, so the negative impact of technological level on environmental pollution is obvious. Industrial structure and environmental pollution also show a significant negative correlation at the significance level of 1%. In other words, the higher proportion of the secondary industry implies the lower degree of environmental pollution. The reasonable explanation for this result is that in the regions with a higher proportion of the secondary industry, the common people have higher demands for environment and health, while the local governments pay more attention to environmental protection and governance, and implement the stricter regulations on work conditions of producers. Therefore, the market has higher barriers to entry. Moreover, the regions with a higher proportion of the secondary industry have achieved a larger output value of the secondary industry, which means that the pollution degree of the unit output value to the environment is relatively low. Both threshold regression results and FE regression results show that environmental regulation has a positive effect on environmental pollution at the confidence level of 95%, which goes contrary to theory. The possible reason is that the high degree of environmental pollution has led to greater efforts in environmental governance in the region, which is manifested in increased environmental supervision. In other words, it is high pollution that leads to strong regulation rather than strong regulation that inhibits high pollution. There is a reverse causality in this regard.

As for other control variables, according to the three regression results, technological level has a significant negative effect on environmental pollution at the significance level of 1%. In other words, since green production has become the mainstream of contemporary society, the high-tech field shows a low degree of environmental pollution. Moreover, the advancement of production technology cannot only greatly improve the economic benefits of the enterprises, but also improve the utilization rate of resources. If an enterprise prioritizes technological innovation, its economic efficiency and corporate image will be greatly embellished. As a result, the use of new technologies has attracted peer enterprises. The spillover effect of technology has prompted enterprises to successively introduce green and efficient technologies, thereby reducing regional environmental pollution. Technological advancement can also improve the regional environmental governance capabilities. Enterprises and local governments prefer centralized environmental governance, so the negative impact of technological level on environmental pollution is obvious. Industrial structure and environmental pollution also show a significant negative correlation at the significance level of 1%. In other words, the higher proportion of the secondary industry implies the lower degree of environmental pollution. The reasonable explanation for this situation is that in the regions with a higher proportion of the secondary industry, the common people demands higher for environment and health, while the local governments pay more attention to environmental protection and governance, and implement stricter regulations on the work conditions of producers. Therefore, the market has higher barriers to entry. Moreover, the regions with a higher proportion of the secondary industry have achieved a larger output value of the secondary industry, which means that the pollution degree of the unit output value to the environment is relatively low. Both threshold regression results and FE regression results prove that environmental regulation has a positive effect on the environmental pollution at the confidence level of 95%, which goes contrary to theory. The possible reason is that the high degree of environmental pollution has led to greater efforts in environmental governance in the region, which is manifested in increased environmental supervision. In other words, it is high pollution that leads to strong regulation rather than strong regulation inhibits high pollution. There is a reverse causality in this regard. 1. Variable selection and data source:

### Empirical Analysis for Measuring Mediating Effect of Environmental Pollution on Public Health

The occurrence of respiratory diseases and lung diseases is largely affected by environmental pollution (47). Practical data also prove that the death rate of respiratory diseases and lung diseases will increase in areas with serious environmental pollution. On the one hand, the development of industrial agglomeration can promote the benign development of local environment, and provide a good living environment for people. Per contra, it worsens the local environment and has a negative impact on the health of the people. Therefore, in judging whether industrial agglomeration can significantly affect public health, the degree of environmental pollution obviously plays a mediating role. Industrial agglomeration indirectly affects the level of public health through environmental pollution. This paper constructs the intermediary effect path according to Baron and Kenny (48) procedures, and investigates the intermediary effect of environmental pollution on the impact of industrial agglomeration on public health.

As an important statistical concept, mediator effect refers to the influence relationship between variables where (X → Y), is not a direct causal chain, but an indirect influence through one or more variables (M). Currently, we call M as a mediator variable, and the indirect influence of X on Y through M is a mediating effect. In this paper, we will draw attention on the intermediary effect test procedure proposed by Wen and Ye (49) to test whether environmental pollution plays an intermediary effect between industrial agglomeration, foreign direct investment, and public health. The specific test steps are as follows: (1) Centralize all variables (make the mean value of variables equal to 0) (2); Test whether the regression coefficient of independent variables (industrial agglomeration and FDI) to dependent variables (public health) is significant; if it is significant, the third step will be carried out, otherwise, the test will be stopped; (3) Baron and Kenny (48) test is used to test the regression coefficients of independent variables (industrial agglomeration and FDI) and intermediary variables (environmental pollution), and whether the regression coefficients of intermediary variables (environmental pollution) and dependent variables (public health) are significant. If both the above coefficients are significant, it means that at least a part of the influence of independent variables on dependent variables is realized by intermediary variables. If at least one is not significant, because the test is less effective (the possibility of making the second type of error is relatively high), it is impossible to conclude, go to step 4. (4) If the Sobel test is significant, it means that the mediating variable plays a mediating role between the independent variable and the dependent variable. Otherwise, there is no mediating effect.

For the mediating effect test, the simulation study found that the strength of Sobel method was higher than that of the sequential regression coefficient method. In other words, Sobel can test more mediating effects than the latter.

(26) $$\begin{array}{l} z=\frac{â\hat{b}}{S_{ab}} \end{array}$$

Where â and $\hat{b}$ are the estimates of *a*and *b*, respectively; the standard error of $\hat{ab}$ is $se\left(ab\right)=\sqrt{{â}^{2}se_{b}^{2}+{\hat{b}}^{2}se_{a}^{2}}$. Here *se*_*a*_ and *se*_*b*_ are the standard errors of *a* and *b*, respectively. In order to test the mediating effect of environmental pollution, we first regressed all the samples, and the regression model is shown in Equations (27) to (29):

(27) $$\begin{array}{l} puh=\beta_{0}+\beta_{1}agg+\beta_{2}fdi+\beta_{3}agg\cdot fdi+\beta_{4}tec+\beta_{5}reg\\ \;+\beta_{6}str+\theta \end{array}$$

(28) $$\begin{array}{l} con=\beta_{0}+\beta_{1}agg+\beta_{2}fdi+\beta_{3}agg\cdot fdi+\beta_{4}tec+\beta_{5}reg\\ \;+\beta_{6}str+\theta \end{array}$$

(29) $$\begin{array}{l} puh=\beta_{0}+\beta_{1}agg+\beta_{2}fdi+\beta_{3}con+\beta_{4}agg\cdot fdi+\beta_{5}tec+\beta_{6}reg\\ \;+\beta_{7}str+\theta \end{array}$$

First, the variables are decentralized, and then regression analysis is performed on models (27), (28), and (29). Table 5 reports the test results of mediating effect of the whole sample. Individual effect and time effect are considered in all the regression models, and the error terms are adjusted by clustering.

**Table 5:** Regression results of mediating effect of environmental pollution.

	**(1)** **puh**	**(2)** **con**	**(3)** **puh**
agg	−0.8827^***^ (0.0024)	−0.3148^***^ (0.0023)	−0.1652^***^ (0.0003)
fdi	−1.3562^***^ (0.0012)	−0.1504^**^ (0.0008)	−0.9237^***^ (0.0014)
con			0.3569^***^ (0.0001)
agg*fdi	−4.2961^**^ (0.0007)	−3.5207^**^ (0.0017)	−3.9418^***^ (0.0009)
tec	−2.6195^***^ (0.0006)	−1.2855^***^ (0.0000)	−2.9481^***^ (0.0172)
reg	−3.0527^**^ (0.0011)	−0.2272^***^ (0.0062)	−3.7041^**^ (0.0000)
str	2.8717^**^ (0.0002)	0.5400^*^ (0.0236)	1.7520^***^ (0.0186)
_cons	2.6934 (0.0015)	0.2451 (0.0244)	1.6725^*^ (0.1042)
Industry	control	control	control
Year	control	control	control
adj. R^2^	0.1926	0.1823	0.2017

^***^, ^**^, and ^*^are significant at the level of 1, 5, and 10% respectively.

The regression results in Table 5 show that industrial agglomeration, FDI, the interaction of industrial agglomeration and FDI, technological progress, and environmental regulation have a significant negative effect on environmental pollution, and have a significant impact on public health in the same direction. The mediating effect of environmental regulation is obvious. In the intermediate effect test, most of the prevariable coefficients are significant. To judge the intermediate effect of environmental pollution more accurately, Sobel test is carried out in this paper. The results show that the development of industrial agglomeration and FDI can improve the level of public health by reducing environmental pollution.

In addition, to test the robustness of the regression results of the mediating effect, this paper uses the number of patent applications as the index to measure technological progress, and the environmental regulation laws and regulations issued by the government as the index to measure the degree of environmental regulation, and then regresses the three models. The regression results are basically consistent with the results in Table 5.

### Robustness Test

To prove the reliability and non-randomness of the threshold regression results, this paper conducts a robustness test. The specific method is explained as follows: The measurement of the environmental regulation of the control variables is expressed as the proportion of the amount of investment in place for industrial pollution control in the industrial added value. The panel threshold regression of the robustness test model (22) is performed, and the regression results are as shown in Table 6. The model still exhibits a dual threshold effect. When the level of FDI is at the left of the first threshold, between the first and the second thresholds and at the right side of the second threshold, the industrial agglomeration coefficient values are all negative and found to be −1.92, −2.61, and −1.98, respectively, and the variation trend is consistent with the previous threshold regression results in the industrial agglomeration in FDI. Among other control variables, technology level and industrial structure coefficient are both negative values, and both are significant when the significance level is 1%. This indicates a positive environmental impact in terms of technology level and industrial structure, similar to the estimation results as shown in Table 4. When the measurement index of a certain control variable is altered, the coefficient signs of explanatory variables do not change. This indicates that the model has obvious robustness.

**Table 6:** Robustness test.

**Variable**	**Coefficient estimate**	***T*-value**
lnagg^*^I(q≦γ1)	−1.9245^***^	−7.8636
nagg^*^I(γ1 < q≦γ2)	−2.6178^***^	−8.0933
nagg^*^I(q>γ2)	−1.9835^***^	−6.1848
lntec	−0.7678^***^	−11.3619
lnstr	−1.4611^***^	−7.9383
lnreg	0.1364^***^	6.9162

^***^, and ^*^are significant at the level of 1, and 10% respectively.

## Discussion and Conclusion

This paper uses the data of seven representative polluting industries of the manufacturing industries of China from 2004 to 2017 to analyze the impact of manufacturing industrial agglomeration on environmental pollution, and further examines the role played by FDI in this process. Based on the theoretical model, the panel threshold regression measurement model proposed by Hansen is adopted, and the empirical research is carried out with foreign capital as the threshold variable. According to the results, in general, under different levels of foreign investment, industrial agglomeration has a restraining effect on environmental pollution, but the degree of restraint is different. Specifically, the positive effect of industrial agglomeration on environment tends to initially increase and subsequently decrease as FDI rises. When the level of FDI is between the first threshold and the second threshold, the maximum inhibitory effect of industrial agglomeration on environmental pollution can be achieved. The technological level and industrial structure of the agglomeration areas have negative impact on the environmental pollution. Environmental regulations and environmental pollution are positively correlated, but they are of little significance. These findings reveal an important transmission mechanism between economic growth and public health and make theoretical contributions to related fields.

Further, the development of industrial agglomeration and FDI can improve the level of the public health by reducing environmental pollution. In the long run, industrial agglomeration can effectively alleviate the environmental pressure through the positive externality of agglomeration. The entry of FDI brings innovations in knowledge, technology, management methods, etc., and promotes the development of production processes in an environmentally friendly manner. The emergence of environmental pollution has seriously threatened the public health. The impact on public health can be expressed through the environment, and the increase of environmental pollution leads to a lower level of public health.

On the one hand, these findings reveal an important transmission mechanism between economic growth and public health, and make theoretical contributions to related fields. On the other hand, these findings also provide policy recommendations for promoting the coordinated development of the economy and environment of China and improving the public health.

We conclude that industrial agglomeration can help curb environmental pollution. Enterprises should make full use of the advantage of agglomeration, organically integrating the input and output of upstream and downstream resources and reduce resource waste. Local governments should spare no efforts to guide enterprises to strive for industrial agglomeration, alleviate the pressure of economic development on the environment, and improve the public health. In addition, when reviewing the qualifications of foreign investors, local governments should say no to foreign companies with serious pollution and high energy consumption (50). The development of the local economy should not be at the expense of the ecological environment and public health. Local governments should strengthen the local environmental supervision, which is the long-term strategy of local economic development. This is a major event related to the health and quality of the life of the local people (51). Slack environmental regulation merely generates temporary economic benefits. On the development road of “post-pollution treatment,” losses exceed gains.

Of course, it is no doubt that our theoretical and empirical analyses are not complete. Firstly, the mathematical model uses production function to analyze the effects of industrial agglomeration and FDI, which does not include other possible mechanisms through which these effects appear. Future research needs to extend the scope of analyses, such as how R&D investment affects the firm environmental performance measured by energy and carbon emissions intensities, etc (52). Secondly, our theoretical analysis takes an assumption of enough low or zero transaction cost, to integrate the externality into production decision making of an enterprise. The situation of high transaction cost is worth paying more attention to in future research. Finally, there are many macro and micro factors affecting the quality of the environment and public health, but this paper cannot cover all possible variables. We hope other scholars continue to incorporate other interesting variables into empirical model in the light of the logic of this research in future.

## Data Availability Statement

The original contributions presented in the study are included in the article/supplementary material, further inquiries can be directed to the corresponding author/s.

## Author Contributions

S-JL: conceptualization, methodology, and software. BS: data curation and writing the original draft. D-XH and W-JJ: regression and writing the original draft. YJ: writing, reviewing, and editing. All authors contributed to the article and approved the submitted version.

## Conflict of Interest

The authors declare that the research was conducted in the absence of any commercial or financial relationships that could be construed as a potential conflict of interest.

## Publisher's Note

All claims expressed in this article are solely those of the authors and do not necessarily represent those of their affiliated organizations, or those of the publisher, the editors and the reviewers. Any product that may be evaluated in this article, or claim that may be made by its manufacturer, is not guaranteed or endorsed by the publisher.
